# Oxylipin composition of high-density lipoprotein is altered in men and Hispanic adults with mild cognitive impairment

**DOI:** 10.1016/j.jlr.2026.101021

**Published:** 2026-03-12

**Authors:** Julia C. Kelliher, Christopher G. Engeland, Jennifer E. Graham-Engeland, Mindy Katz, Claudette Kessler, Gregory C. Shearer

**Affiliations:** 1 Integrative and Biomedical Physiology, The Pennsylvania State University, University Park, PA, USA; 2 Department of Biobehavioral Health, The Pennsylvania State University, University Park, PA, USA; 3 Ross and Carol Nese College of Nursing, The Pennsylvania State University, University Park, PA, USA; 4 Einstein Aging Study, Albert Einstein College of Medicine, Bronx, NY, USA; 5 Nutritional Sciences Department, The Pennsylvania State University, University Park, PA, USA

**Keywords:** oxylipins, high-density lipoprotein, HDL, mild cognitive impairment, MCI, Alzheimer's disease, AD, dementia, sex differences, racial/ethnic differences, lipids, fatty acids, oxidized fatty acids, PUFA, metabolism, lipoxygenase, cytochrome p450 epoxygenase, omega 3, EPA, DHA, HEPE, HDoHE, HDHA

## Abstract

High-density lipoprotein (HDL) oxylipins are potent inflammatory mediators. HDL dyshomeostasis and inflammation increase mild cognitive impairment (MCI) and dementia risk, which vary by gender and race/ethnicity. It is unknown whether and how HDL oxylipin profiles differ between non-MCI and MCI individuals, or if potential differences are gender- and/or race/ethnicity dependent. In this targeted lipidomics study, we profiled plasma HDL oxylipins in older (70+) adults (N = 222) with or without MCI to determine how HDL oxylipin composition relates to cognitive impairment status. HDL oxylipin concentrations were analyzed by cognitive status, gender, and race/ethnicity (non-Hispanic Black, Hispanic, and non-Hispanic white). We found a gender- and race/ethnicity-specific association between MCI and lower HDL oxylipin content, which was independent of overall HDL-c concentrations. The HDL of MCI men contained lower amounts of anti-inflammatory and vasodilatory omega (ω)3 EPA C20:5ω3-derived hydroxyeicosapentaenoic acids (HEPEs) and DHA C22:6ω3-derived hydroxydocosahexaenoic acids than that of non-MCI men. Similarly, Hispanic participants with MCI had lower HDL concentrations of EPA C20:5ω3-derived HEPEs and DHA C22:6ω3-derived hydroxydocosahexaenoic acids than non-MCI Hispanic participants. Higher HDL concentrations of EPA C20:5ω3-derived HEPEs appeared protective against MCI in both men and Hispanic individuals. Further, higher oxylipin concentrations within HDL correlated with better cognition in non-Hispanic white women. This work identifying altered HDL oxylipin composition in MCI highlights a novel dysregulated lipid signaling pathway in cognitive decline. Reduced anti-inflammatory and vasodilatory ω3 oxylipins within HDL in MCI men and Hispanic individuals provide molecular evidence linking together HDL functionality, inflammation, and dementia risk.

Oxylipins are potent oxygenated polyunsaturated fatty acid (PUFA)-derived metabolites that mediate the biological effects of PUFAs to regulate inflammation and vascular homeostasis (1, 2, 3). Excessive inflammation, lipid peroxidation, oxidative stress, and vascular dysfunction are key contributors to the development of cognitive impairment and dementia (4, 5, 6, 7, 8). Dementia is a global public health concern projected to afflict over 152 million people by 2050 and most commonly caused by Alzheimer’s disease (AD) and related dementias (ADRD) (9, 10). AD prevalence varies by gender and race/ethnicity, where women, non-Hispanic Black individuals, and Hispanic individuals have higher rates of AD compared to men and non-Hispanic white individuals, respectively (11). Over 99% of clinical trials focused on protein pathologies in AD have failed, and novel treatment strategies targeting lipid dysfunction in AD may help reduce AD incidence and burden (12, 13, 14, 15).

The brain is the second most lipid-rich organ in the body after adipose tissue, and abnormal lipid granules were identified in the first recorded case of AD by Alois Alzheimer in 1906 (13, 16). The brain is highly enriched in PUFAs, particularly the omega (ω)3 PUFA docosahexaenoic acid (DHA) C22:6ω3 and ω6 PUFA arachidonic acid (AA) C20:4ω6, which maintain important roles in physiological brain functions, like neuroplasticity, neurotransmission, and membrane fluidity (17, 18). AD risk is modulated by both high-density lipoprotein (HDL) lipid transport functionality and by dietary lipid intake. The strongest known genetic risk factor for AD, the E4 isoform of the *APOE* gene encoding apolipoprotein(apo)E, significantly impairs lipid uptake by HDL and reduces amyloid beta clearance from the brain (19, 20, 21, 22). In contrast, higher concentrations of plasma apoA-I HDL and HDL-cholesterol (HDL-c) support HDL’s lipid-trafficking abilities and reduce the risk of dementia and mild cognitive impairment (MCI), the first symptomatic stage of AD where people experience abnormal cognitive impairment at a level that does not significantly interfere with their daily lives (23, 24, 25, 26). Higher intake of ω3 PUFAs like eicosapentaenoic acid (EPA) C20:5ω3 and DHA C22:6ω3 via dietary consumption or ω3 PUFA supplementation increases ω3 PUFA-derived oxylipin concentrations and is associated with reduced AD risk, which may be more pronounced in apoE4 non-carriers (27, 28, 29, 30, 31, 32, 33, 34). Conversely, lower PUFA intake, higher saturated fatty acid consumption, and dyslipidemia during midlife are associated with higher AD risk (27, 30, 31, 32, 33, 34, 35). Hispanic individuals, who have a higher risk and incidence of MCI and AD, have lower HDL and ω3 PUFA concentrations on average compared to non-Hispanic white individuals (11, 36, 37, 38, 39). Although HDL and PUFA intake are known to affect dementia risk, it is not well understood how the HDL composition of PUFA-derived oxylipins is altered during the early stages of cognitive decline.

HDL transports bioactive oxylipins that are potent regulators of inflammation and vascular function (40, 41, 42, 43). The majority of plasma oxylipins are esterified in HDL, supporting rapid release and sequestration of nonesterified oxylipins to modulate oxylipin bioactivity (2, 44, 45). Oxylipins have diverse biological effects (2, 3). For example, AA C20:4ω6-derived 5-hydroxyeicosatetraenoic acid (5-HETE) and 12-HETE stimulate neutrophil chemotaxis, whereas EPA C20:5ω3-derived 12-hydroxyeicosapentaenoic acid (12-HEPE) and DHA C22:6ω3-derived 17-hydroxydocosahexaenoic acid (17-HDoHE) exert vasodilatory and anti-inflammatory effects (46, 47, 48, 49, 50). Oxylipin profiles are affected by diet, sex, aging, and disease (51, 52, 53, 54, 55). Non-esterified oxylipin profiles in plasma and cerebrospinal fluid are altered in people with MCI and AD, and esterified brain oxylipin concentrations are affected in rodent AD models (56, 57, 58, 59, 60). Further, oxylipin biosynthetic enzymes like lipoxygenases (LOX), are emerging therapeutic targets for AD (61). Whereas oxylipin metabolism and HDL lipid transport appear to be dysregulated during MCI and AD, it is unknown whether HDL oxylipin profiles differ in people with or without MCI or AD.

Impaired lipid transport by HDL is a well-established risk factor for MCI and AD (25, 62, 63). Yet, studies to date have not characterized the composition of bioactive oxylipins in HDL in individuals with or without MCI or AD. Gaining a better understanding of oxylipin profile changes within HDL in subjects with or without MCI could biologically link the aforementioned impact of oxylipin precursor PUFAs and HDL on dementia risk to previously identified aberrant plasma oxylipin profiles in subjects with AD. To the best of our knowledge, this targeted lipidomics study is the first to *1*) evaluate the relationship between HDL oxylipin content and MCI, *2*) quantify total (non-esterified + esterified) oxylipins in individuals with or without MCI, and *3*) assess gender- and racially/ethnically-dependent oxylipin profile differences in people with or without MCI. We explored if and how concentrations of a panel of 59 total (non-esterified + esterified) plasma HDL oxylipins differed by participant cognitive impairment status in racially/ethnically diverse older men and women, as assessed by MCI status and Montreal Cognitive Assessment (MoCA) scores. We hypothesized that *1*) people with MCI would have a more pro-inflammatory oxylipin profile within HDL compared to people without MCI, *2*) non-Hispanic Black and Hispanic participants with MCI, who are at greater risk of progressing to AD, would have lower HDL oxylipin concentrations than white participants with MCI and non-Hispanic Black and Hispanic participants without MCI, and *3*) that HDL oxylipin concentrations would positively correlate with participant MoCA score. Overall, this research assessing HDL oxylipin profile differences in older non-MCI and MCI adults could facilitate the identification of novel therapeutic targets and prevention strategies for reducing cognitive impairment incidence and progression to dementia.

## Materials and methods

### Study design

**Fig. 1:**
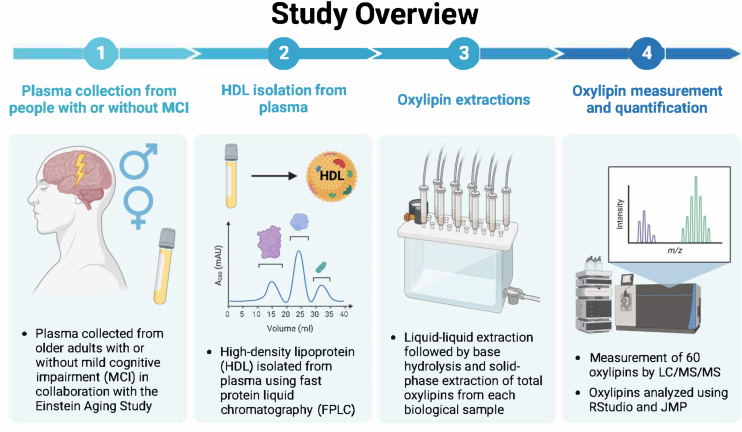
HDL oxylipins were measured in older adults with or without MCI. Total (non-esterified + esterified) oxylipins in HDL-rich plasma were quantified from older adults (N = 222) with or without MCI.

A panel of 59 oxylipins was measured and analyzed from plasma HDL of men and women 70+ years old who were clinically classified as non-MCI or MCI individuals (Fig. 1).

### Subjects

This study was approved by the Albert Einstein College of Medicine Institutional Review Board and the Pennsylvania State University Institutional Review Board. All human-subjects research was conducted in accordance with the Declaration of Helsinki. Participants (N = 222; 146 women/76 men) were systematically recruited from the Bronx, New York, USA as part of the Einstein Aging Study (EAS) based at the Albert Einstein College of Medicine and gave informed consent for participation in this study. Collection of participant plasma and data for this study occurred between September 2017 and January 2020, and plasma processing for oxylipin quantification occurred an average of 1.5 years after plasma collection. Body mass index (BMI) was calculated according to participant height and weight (kilogram/meter^2^). Participants underwent extensive neurocognitive assessments and MCI screening according to Jak/Bondi criteria that used an algorithm to assess impairment in five different neurocognitive domains: memory, language, executive function, attention, and visuospatial abilities (64, 65, 66). Cognitively impaired scores were defined as being greater than one standard deviation from the age-corrected normative mean (65). Accordingly, participants were considered to have MCI if they had impaired scores on two assessments within at least one neurocognitive domain, if they had impaired scores in one assessment across three different neurocognitive domains, or if they had functional inability on all four items (ability to use the telephone, ability to travel independently, ability to be responsible for medications, and ability to handle finances) on the Lawton/Brody Instrumental Activities of Daily Living Scale that indicated a reduced ability to function independently in daily activities (64, 65, 67). Other neurocognitive assessments were administered to study participants as part of the EAS battery, including the Montreal Cognitive Assessment (MoCA), which is a 30-item, highly sensitive screening tool for cognitive impairment, where participants receive a score between 0 and 30, with 30 representing the best possible score of assessed cognitive performance (68). Participants additionally completed demographic psychosocial questionnaires that included the short form Geriatric Depression Scale (GDS). The GDS is a 15-item, self-reported screen for depression in older adults where participants receive a score between 0 and 15, with 15 representing the worst possible score of self-reported depressive symptomatology (69).

### Clinical lipid panel

Triglycerides, total cholesterol, HDL-c, and low-density lipoprotein-cholesterol (LDL-c) were quantified from fasted participant plasma. Triglycerides and total cholesterol were quantified using standard colorimetric assays. HDL-c was measured using a two-step homogenous cholesterol oxidase method on the Abbott Alinity Platform. LDL-c was calculated using total cholesterol, HDL-c, and triglyceride concentrations according to the Friedewald equation (70). Clinical lipid panel data were not collected for nine participants (eight women/one man).

### Plasma HDL isolation for HDL oxylipin quantification

Blood was collected from all fasted study participants (N = 222) in ethylenediaminetetraacetic acid (EDTA)-coated tubes, which is the most stable storage form for plasma oxylipins and reduces artificial oxylipin production during sample storage (71, 72, 73). Blood was centrifuged for 15 min at 1,500 *g* for plasma isolation, and plasma was stored at −80°C for an average of 1.5 years until samples were processed for oxylipin quantification. HDL was isolated from plasma using fast protein liquid chromatography (FPLC, AKTApurifier, GE, Amersham Biosciences, Sweden) on a conditioned Superose 6 Increase 10/300 GL size-exclusion column (Cytiva, MA) with a degassed 0.9% sodium chloride (pH 7.4) mobile phase containing 0.1 g/l EDTA and 0.1 g/l butylated hydroxytoluene antioxidants, as previously described in numerous prior publications (74, 75, 76). HDL-rich plasma fractions for each participant were pooled together and immediately frozen at −80°C until further processing for oxylipin extractions. All FPLC chromatograms were visually inspected to ensure that there was no drift in lipoprotein elution times, and potential effects of FPLC column run-to-run variability on experimental results were minimized by using an MCI status- and gender-balanced sample batching strategy for the sample order of HDL isolation.

### Oxylipin extractions

Total (esterified + non-esterified) oxylipins, the most stable form of plasma oxylipins, were extracted from plasma HDL, as previously described, using a protocol that is consistent with published best practices for plasma oxylipin quantification (71, 76, 77). Briefly, a surrogate solution with known concentrations of deuterated lipid standards (Cayman Chemical, MI) that represented each chemical class of quantified oxylipins and an antioxidant mixture of 0.2 mg/ml butylated hydroxytoluene and EDTA was added to each plasma HDL sample immediately prior to oxylipin extraction. Samples were then subjected to liquid-liquid extraction using the organic solvents isopropyl alcohol, cyclohexane, and ammonium acetate to isolate the lipid portion of each sample. Lipid extracts were dried by nitrogen gas, reconstituted in methanol and toluene, and subjected to base hydrolysis using sodium methoxide, heat, and water. Total (esterified + non-esterified) oxylipins were extracted from total lipid extracts using solid-phase extraction on hydrophilic-lipophilic balanced columns (Macherey-Nagel, PA) with the organic solvents methanol, ethyl acetate, and acetic acid. Eluted oxylipins were dried with nitrogen gas.

### LC/MS/MS oxylipin measurements

Targeted liquid chromatography with tandem mass spectrometry (LC/MS/MS) was used to measure a panel of 59 total oxylipins from each biological sample, as previously described (76). Prior to injection on the LC/MS/MS, dried samples of extracted oxylipins were reconstituted in LC/MS-grade methanol that contained the internal standard 12-cyclohexylamino-carbonyl-amino-dodoecanoic acid to account for LC/MS/MS machine variability, and 5 μl of each sample was injected on the Xevo TQD Triple Quadrupole (Waters, MA). Samples were separated using LC with a 15-min gradient run of two solvents (solvent A: 0.1% acetic acid in water; solvent B: 90:10 acetonitrile:isopropanol) on a CORTECS C18+ column (Waters, MA) at a flow rate of 500 μl per minute. Following physical separation of samples via LC, samples were ionized with electrospray ionization in negative ion mode, and a targeted approach with multiple reaction monitoring was used to detect a panel of 59 distinct oxylipins for each biological sample. The cone voltage and collision energy for each oxylipin were previously determined by injecting individual pure oxylipin standards (Cayman Chemicals, MI) into the LC/MS/MS and utilizing Waters IntelliStart software to identify settings that enable optimal detection of each oxylipin. Pure oxylipin standards (Cayman Chemicals, MI) for each of the 59 measured oxylipins were run prior to each LC/MS/MS batch of samples for confirmation of accurate detection of each oxylipin and for the generation of calibration curves for calculating oxylipin concentrations. Oxylipin peaks were selected using TargetLynx software (Waters, MA), and each chromatogram was visually inspected for accuracy before exporting peak areas for oxylipin concentration calculations.

### Oxylipin concentration calculations

Data analysis was performed using Excel, R Studio (2022.12.0+353), and JMP Pro (16.2.0). Peak areas of oxylipins detected using LC/MS/MS were divided by the peak area of 12-cyclohexylamino-carbonyl-amino-dodoecanoic acid for each sample. Oxylipin calibration curves that contained five different known concentrations of each pure oxylipin standard per curve were fit with power regression models to calculate experimental oxylipin concentrations. Calculated oxylipin concentrations were adjusted by plasma sample volume injected on the FPLC and percent recovery of deuterated lipid standards for each sample to enable reporting of nanomolar concentrations of oxylipins within HDL plasma. Participant oxylipin concentrations were not adjusted by HDL-c concentrations because HDL-c concentrations did not differ by cognitive status. Adjusting oxylipin concentrations by HDL-c concentrations did not alter our experimental results, and providing direct concentrations of HDL oxylipins in plasma has value for the interpretation of the biological significance of reported values.

### Statistical analysis

Our primary analytical goal was to compare HDL oxylipin profiles in participants without indication of MCI (“non-MCI” participants) to those with indication of MCI (“MCI” participants). We were also interested in how oxylipin differences between non-MCI and MCI participants varied by gender and race/ethnicity. Calculated oxylipin concentrations were logarithm base 10 transformed prior to further analysis to normalize the data distribution. Outlier sensitivity analyses and leverage plots in JMP were utilized to identify outliers prior to all performed analyses. Outlier tests on the dataset as a whole identified one participant (a non-MCI, Black woman) as an outlier, and this participant was removed from the study. For stratified data analyses, outliers that biased results toward positive findings were removed from the analysis. Because oxylipin biological function can be broadly categorized by their precursor PUFA (e.g., AA vs. DHA), pathway of production (e.g., lipoxygenase [LOX] vs. cytochrome p450 [CYP]), and functional group chemistry (e.g., alcohol vs. epoxide), the analyzed oxylipins were grouped by precursor PUFA, functional group chemistry, and biosynthetic pathway of production unless otherwise specified in text (2).

We used mixed-model analysis of variances with unequal variances to assess grouped HDL oxylipin concentration differences between ethnically/racially diverse men and women with or without MCI. Fixed effects used in these models were MCI status, gender, race/ethnicity, metabolite, extraction batch, and full factorial interactions between all factors. Non-significant interaction terms were removed from each model to maximize parsimony and reduce Bayesian information criterion scores.

To explore early HDL oxylipin changes in an even more subtle cognitive impairment phenotype, we further assessed HDL oxylipin concentrations in non-MCI individuals with a “normal” MoCA score (≥26), non-MCI individuals with an “abnormal” MoCA score (<26), and MCI individuals with an “abnormal” MoCA score (<26). The MoCA score of 26 was used as a cutoff for this analysis based on prior research showing 90% sensitivity of detecting MCI in individuals who had an MoCA score <26 (68, 78). Participants with MCI and an MoCA score ≥26 were excluded from this analysis (four non-Hispanic white women, two non-Hispanic Black women). Fixed effects used in these models were cognitive status (non-MCI with normal MoCA, non-MCI with abnormal MoCA, and MCI with abnormal MoCA), gender, race/ethnicity, metabolites, extraction batch, and full factorial interactions between all factors. Non-significant interaction terms were again removed from each model to maximize model parsimony and reduce model Bayesian information criterion scores. We also conducted correlation analyses between participant HDL oxylipin concentrations and continuous MoCA scores using Spearman rank correlation.

Student’s *t*-tests were used for analyzing post hoc differences in significant (*P* < 0.05) model effects and interactions, where a false discovery rate correction at q = 0.1, unless otherwise specified in text, was applied to account for the number of test comparisons (79). Post hoc Student’s *t*-tests were only conducted for comparisons that differed by one factor (e.g., women without MCI vs. women with MCI) and not for comparisons that differed by more than one factor (e.g., women without MCI vs. men with MCI). Results are presented as mean nanomolar oxylipin concentrations ± the 95% confidence interval (CI). For all graphs, blue bars represent non-MCI participants, and orange bars represent MCI participants.

## Results

### MCI participants had lower MoCA scores, fewer years of formal education, and higher GDS scores

Study participants were 161 non-MCI and 61 MCI diverse older adults (N = 222; 40% non-Hispanic Black, 13% Hispanic, 47% non-Hispanic white) who were two-thirds women and had a mean age of approximately 77 years old (Table 1). Consistent with higher MCI risk in people who are non-Hispanic Black and Hispanic, there was a greater proportion of MCI participants who were non-Hispanic Black or Hispanic compared to non-MCI participants (80). All participants were at least 70 years of age (range: 70.4–90.6 years old). Non-MCI and MCI participants did not have significant differences in mean age (Table 1, *P* = 0.2). As expected, MoCA scores were lower in MCI participants compared to non-MCI participants (Table 1, *P* < 0.0001) (68). In line with known protective effects of education on MCI development, MCI participants also had fewer years of education compared to non-MCI participants (Table 1, *P* = 0.01) (81). MCI participants had higher GDS scores than non-MCI participants (Table 1, *P* = 0.03). Non-MCI and MCI participants did not have significantly different BMI (Table 1, *P* = 0.9), triglycerides (Table 1, *P* = 0.8), total cholesterol (Table 1, *P* = 0.6), HDL-c (Table 1, *P* = 0.3), or LDL-c (Table 1, *P* = 0.97) concentrations. There were significant effects of gender on participant total cholesterol (Table 1, *P* = 0.0002), HDL-c (Table 1, *P* < 0.0001), and LDL-c (Table 1, *P* = 0.03) concentrations. There was a significant effect of race/ethnicity on triglyceride concentrations (Table 1, *P* = 0.009), where non-Hispanic Black participants had reduced triglyceride concentrations compared to non-Hispanic white participants (*P* = 0.03) and compared to Hispanic participants (*P* = 0.04), regardless of MCI status.

**Table 1:** Participant demographics

MCI status	Non-MCI: 161 (73%)			MCI: 61 (27%)		
Gender	Women: 107 (66%)	Men: 54 (34%)	Collapsed	Women: 39 (64%)	Men: 22 (36%)	Collapsed
Ethnicity/race	B: 49 (46%)	B: 10 (19%)	B: 59 (37%)	B: 18 (46%)	B: 12 (55%)	B: 30 (49%)
	H: 11 (10%)	H: 6 (11%)	H: 17 (10%)	H: 10 (26%)	H: 2 (9%)	H: 12 (20%)
	W: 47 (44%)	W: 38 (70%)	W: 85 (53%)	W: 11 (28%)	W: 8 (36%)	W: 19 (31%)
Age (years)	77.5 (76.6, 78.4)	76.0 (74.8, 77.3)	76.7 (76.0, 77.5)	78.1 (76.6, 79.6)	77.6 (75.6, 79.5)	77.8 (76.6, 79.1)
MoCA	24.8 (24.2, 25.4)	24.3 (23.4, 25.2)	24.5 (24.0, 25.1)^*A*^	20.7 (19.7, 21.7)	21.4 (20.0, 22.8)	21.1 (20.2, 21.9)^*B*^
Education (years)	15.4 (14.8, 16.1)	15.4 (14.4, 16.4)	15.4 (14.8, 16.0)^*A*^	14.2 (13.0, 15.3)	13.8 (12.3, 15.3)	14.0 (13.0, 14.9)^*B*^
GDS	1.92 (1.54, 2.29)	2.17 (1.63, 2.70)	2.04 (1.71, 2.37)^*A*^	2.82 (2.19, 3.45)	2.36 (1.52, 3.20)	2.59 (2.07, 3.12)^*B*^
BMI	29.3 (24.0, 34.6)	29.5 (22.5, 36.5)	29.4 (23.5, 35.3)	30.5 (22.9, 38.0)	27.4 (24.0, 30.7)	29.3 (22.8, 35.8)
Total cholesterol	192.0 (154.3, 229.9)	167.1 (127.0, 207.2)	183.6 (143.3, 223.8)	185.8 (142.9, 228.7)	171.9 (125.2, 218.6)	180.6 (136.2, 225.0)
HDL-c	59.0 (43.2, 74.8)	46.2 (36.2, 56.2)	54.6 (39.3, 70.0)	54.6 (42.0, 67.2)	48.4 (33.3, 63.5)	52.3 (38.5, 66.0)
LDL-c	107.6 (72.0, 143.2)	95.5 (63.4, 127.6)	103.5 (68.7, 138.3)	106.8 (68.8, 144.9)	98.5 (60.4, 136.6)	103.7 (65.8, 141.7)
Triglycerides	109.0 (55.6, 162.5)	111.8 (60.6, 163.0)	110.0 (57.4, 162.5)	110.3 (45.9, 174.7)	104.9 (63.3, 146.5)	108.3 (51.6, 164.9)

The number of study participants (N = 222) with or without MCI is listed in the table, with the proportion of each group relative to the total study sample size shown in parentheses. The number of participants of each gender and ethnicity/race is similarly displayed, with the proportion of each group relative to non-MCI or MCI groups shown in parentheses. Participant mean age in years, MoCA score, education in years, and GDS score are listed with the 95% confidence interval shown in parentheses. Participant mean BMI, total cholesterol, HDL-c, LDL-c, and triglyceride concentrations ± one standard deviation of the mean are shown in parentheses. Significant differences are indicated by differing superscript letters.

B, non-Hispanic Black; H, Hispanic; W, non-Hispanic white.

### Global HDL oxylipin composition differences between non-MCI and MCI participants: men with MCI and Hispanic participants with MCI had lower HDL oxylipin concentrations compared to non-MCI participants

**Fig. 2:**
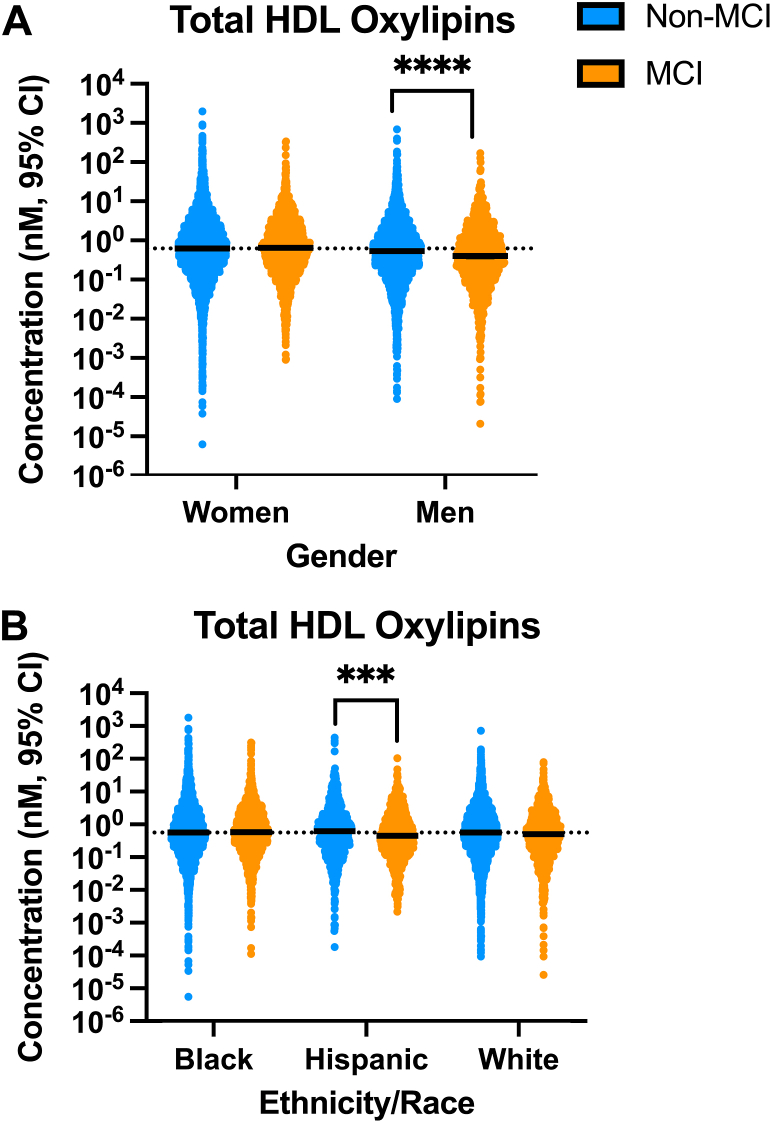
Men with MCI and Hispanic participants with MCI had lower global concentrations of HDL oxylipins. Graphs indicate the nanomolar (nM) oxylipin concentrations with the 95% confidence interval (CI) of global total (non-esterified + esterified) oxylipins in HDL in individuals with or without MCI separated by gender (A) or ethnicity/race (B). Men without MCI: n = 54. Men with MCI: n = 22. Hispanic participants without MCI: n = 17. Hispanic participants with MCI: n = 12. ∗∗∗*P* < 0.001, ∗∗∗∗*P* < 0.0001.

Because HDL transports immunomodulatory oxylipins and because dysfunctional HDL lipid trafficking increases the risk of neuroinflammatory diseases like AD, we hypothesized that the oxylipin content in HDL would differ between individuals with or without MCI (25, 40). We first examined the effects of participant MCI status, gender, and race/ethnicity on global HDL oxylipin content for all total (esterified + non-esterified) quantified oxylipins. There was a significant effect of MCI status (*P* = 0.001), interaction between MCI status and gender (*P* = 0.0006), and interaction between MCI status and race/ethnicity (*P* = 0.01) on HDL oxylipin concentrations. MCI participants had a global reduction in HDL oxylipin content compared to non-MCI participants (Supplemental Fig. S1, *P* = 0.001). Men with MCI had lower HDL oxylipin concentrations compared to men without MCI (Fig. 2A, *P* < 0.0001), and Hispanic participants with MCI had lower HDL oxylipin concentrations compared to Hispanic participants without MCI (Fig. 2B, *P* = 0.0002).

### Functional oxylipin differences in the HDL of non-MCI and MCI participants: anti-inflammatory and vasodilatory ω3 oxylipin concentrations were lower in the HDL of men and Hispanic individuals with MCI compared to non-MCI individuals

**Fig. 3:**
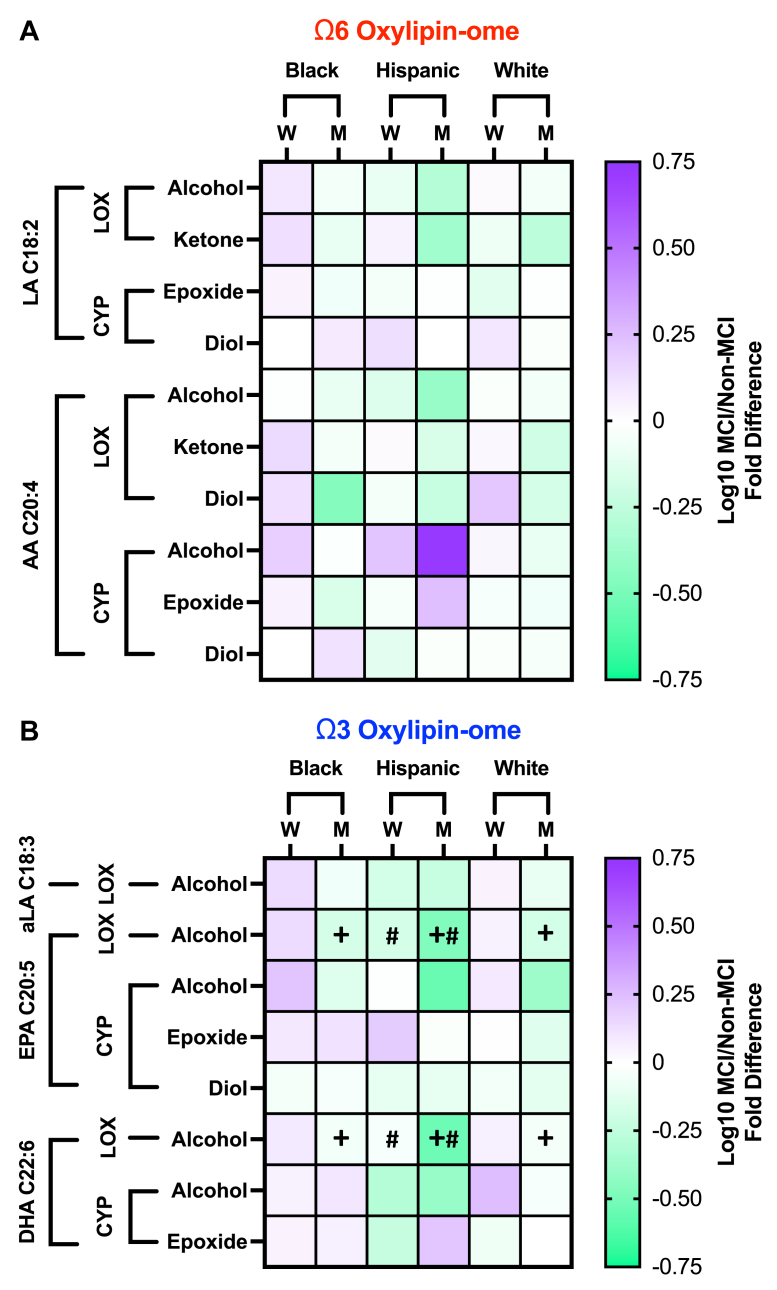
Participants with MCI had distinct HDL oxylipin-omes. Heatmaps show omega (ω)6 (A) and ω3 (B) HDL oxylipin profile shifts in MCI participants (n = 61) compared to the non-MCI participant reference group (n = 161). Purple indicates higher oxylipin concentrations, and green corresponds with lower oxylipin concentrations in the MCI group. Stronger color intensity in the scale bars corresponds with larger fold differences in oxylipin concentrations in the MCI group. The + symbol represents a significant effect of MCI in men collapsed across race/ethnicity, and the $ symbol represents a significant effect of MCI in Hispanic individuals collapsed across gender. Effects that pass false discovery rate correction are considered significant when *P* < 0.05. AA, arachidonic acid; aLA, alpha-linolenic acid; CYP, cytochrome p450; DHA, docosahexaenoic acid; EPA, eicossapentaenoic acid; LA, linoleic acid; LOX, lipoxygenase; M, men; W, women.

**Fig. 4:**
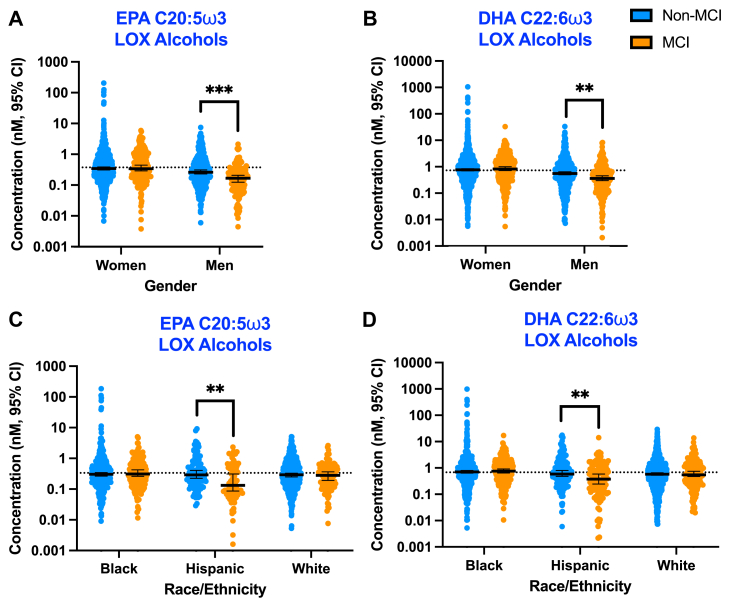
Men with MCI and Hispanic participants with MCI had lower anti-inflammatory omega (ω)3 oxylipins. Graphs indicate the nanomolar (nM) oxylipin concentrations with the 95% confidence interval (CI) of total (non-esterified + esterified) oxylipins in HDL in individuals with or without MCI separated by gender (A and B) or ethnicity/race (C and D). Men without MCI: n = 54. Men with MCI: n = 22. Hispanic participants without MCI: n = 17. Hispanic participants with MCI: n = 12. ∗∗*P* < 0.01, ∗∗∗*P* < 0.001.

To better understand whether the observed gender- and race/ethnicity-dependent loss of HDL oxylipin concentrations in MCI participants was driven by a uniform oxylipin concentration reduction across different classes of oxylipins or by a more selective loss of particular oxylipins, we analyzed how MCI status, gender, and race/ethnicity impacted groups of oxylipins that share similar biological functions. For these analyses, oxylipins were grouped by precursor PUFA (linoleic acid aC18:2ω6, AA C20:4ω6, alpha-linolenic acid C18:3ω3, EPA C20:5ω3, or DHA C22:6ω3), enzymatic pathway of production (LOX/auto-oxidation or CYP epoxygenase), and functional group chemistry (alcohol, diol, epoxide, or ketone). The grouped HDL oxylipin concentration differences between non-MCI and MCI participants are shown qualitatively for all quantified oxylipins in Figure 3 and quantitatively for significant oxylipin concentration differences in Figure 4. Overall, MCI participants had lower concentrations of HDL oxylipins, particularly of ω3 oxylipins, compared to non-MCI participants (Figs. 3 and 4).

We found that the lower global concentrations of HDL oxylipins in Figure 2 in men with MCI and in Hispanic participants with MCI were driven by a loss of EPA C20:5ω3-derived HEPEs and DHA C22:6ω3-derived HDoHEs made through LOX or auto-oxidation pathways of production (Figs. 3, 4, Supplemental Figs. S2 and S3). HEPEs and HDoHEs exert anti-inflammatory and vasodilatory effects and are important regulators of glucose metabolism (46, 47, 48, 49, 82, 83, 84, 85, 86, 87, 88). Collapsed across gender and race/ethnicity, MCI participants had significantly lower concentrations of EPA C20:5ω3/LOX-derived HEPEs in HDL compared to non-MCI participants (*P* = 0.002; Supplemental Fig. S2). There were significant interactions between MCI status and gender for both EPA C20:5ω3-derived HEPEs (*P* = 0.002) and DHA C22:6ω3-derived HDoHEs (*P* = 0.002) synthesized through the LOX enzymatic pathway of production; men with MCI had lower concentrations of EPA C20:5ω3/LOX-derived HEPEs (Fig. 4A, *P* = 0.0002; Supplemental Fig. S3) and DHA C22:6ω3/LOX-derived HDoHEs (Fig. 4B, *P* = 0.001; Supplemental Fig. S3) in HDL compared to men without MCI. There was also a significant interaction between MCI status and race/ethnicity on concentrations of EPA C20:5ω3/LOX-derived HEPEs (*P* = 0.04) and DHA C22:6ω3/LOX-derived HDoHEs (*P* = 0.01); Hispanic participants with MCI had lower concentrations of EPA C20:5ω3/LOX-derived HEPEs (Fig. 4C, *P* = 0.002; Supplemental Fig. S3) and DHA C22:6ω3/LOX-derived HDoHEs than Hispanic participants without MCI (Fig. 4D, *P* = 0.002; Supplemental Fig. S3).

### Individual oxylipin differences between MCI and non-MCI participants: MCI participants had lower HDL oxylipin concentrations across most quantified oxylipins compared to non-MCI participants

**Fig. 5:**
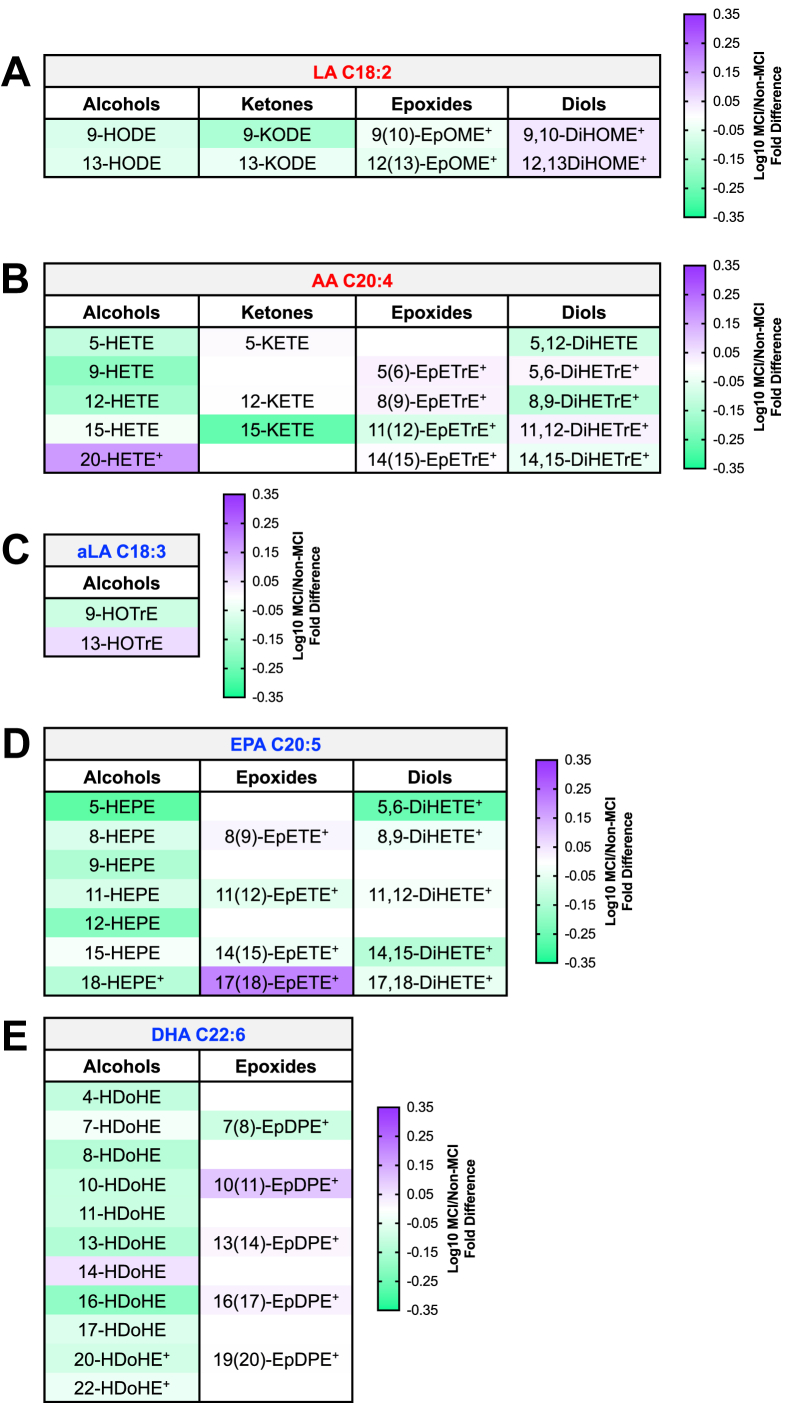
HDL oxylipins were predominantly lower in participants with MCI. Heatmaps show qualitative individual omega (ω)6 (A and B) and ω3 (C–E) HDL oxylipin profile differences in MCI participants (n = 61) compared to the non-MCI participant reference group (n = 161). Oxylipins produced through CYP biosynthetic enzymes are denoted by ^+^; all other oxylipins are made through LOX or auto-oxidation pathways of production. Purple indicates higher oxylipin concentrations, and green corresponds with lower oxylipin concentrations in the MCI group. Stronger color intensity in the scale bars corresponds with larger fold changes in oxylipin concentrations in the MCI group. AA, arachidonic acid; aLA, alpha-linolenic acid; DHA, docosahexaenoic acid; DiHETE, dihydroxyeicosatetraenoic acid; DiHETrE, dihydroxyeicosatrienoic acid; DiHOME, dihydroxyoctadecenoic acid; EPA, eicosapentaenoic acid; EpDPE, epoxydocosapentaenoic acid; EpETE, epoxyeicosatetraenoic acid; EpETrE, epoxyeicosatrienoic acid; EpOME, epoxyoctadecenoic acid; HDoHE, hydroxydocosahexaenoic acid; HEPE, hydroxyeicosapentaenoic acid; HETE, hydroxyeicosatetraenoic acid; HODE, hydroxyoctadecadienoic acid; HOTrE, hydroxyoctadecatienoic acid; KETE, oxoeicosatetraenoic acid; KODE, oxooctadecadienoic acid; LA, linoleic acid.

To validate that individual HDL oxylipins within each oxylipin group reflected grouped HDL oxylipin concentration differences between non-MCI and MCI participants, we qualitatively assessed differences in HDL oxylipin profiles between non-MCI and MCI participants. We found that concentration differences between non-MCI and MCI participants of individual oxylipins that shared the same precursor PUFA, pathway of production, and functional group chemistry predominantly reflected grouped HDL oxylipin concentration differences between non-MCI and MCI participants (Fig. 5). Some exceptions to this consistent pattern were observed for vasoconstrictive AA C20:4ω6-derived 15-oxoeicosatetraenoic acid (15-KETE) and vasodilatory EPA C20:5ω3/LOX-derived 17(18)-epoxyeicosatetraenoic acid (17[18]-EpETE), which may correspond to activation of specific compensatory signaling pathways in individuals with MCI (Fig. 5) (89, 90). Interestingly, MCI participants may have altered AA C20:4ω6 metabolism compared to non-MCI participants; this is seen by non-significantly lower concentrations of pro-inflammatory AA C20:4ω6-derived alcohols made through the LOX enzymatic pathway of production and higher concentrations of vasoconstrictive AA C20:4ω6-derived alcohol 20-HETE made through the CYP pathway of production (Fig. 5) (2, 50, 91).

### HDL oxylipin profile differences based on cognitive impairment severity: Non-MCI men and Hispanic participants with abnormal MoCA scores had higher HDL composition of anti-inflammatory EPA-derived HEPEs than MCI men and Hispanic participants with abnormal MoCA scores

**Fig. 6:**
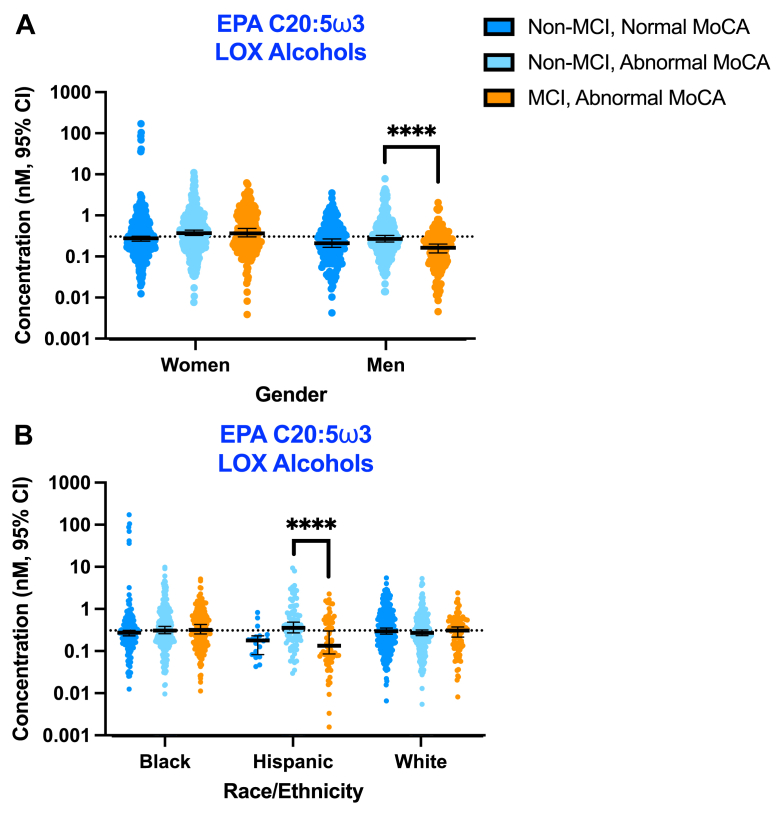
Men and Hispanic participants without MCI but with abnormal MoCA scores had higher anti-inflammatory EPA-derived HEPE concentrations in HDL. Graphs indicate the nanomolar (nM) oxylipin concentrations with the 95% confidence interval (CI) of total (non-esterified + esterified) oxylipins in HDL in individuals with or without MCI separated by gender (A) or ethnicity/race (B). An MoCA score of 26 or above was considered normal. Non-MCI, normal MoCA: n = 73. Non-MCI, abnormal MoCA: n = 89. MCI, abnormal MoCA: n = 55. Men without MCI: n = 54. Men with MCI: n = 22. Hispanic participants without MCI: n = 17. Hispanic participants with MCI: n = 12. ∗∗∗∗*P* < 0.0001.

To better understand changes in HDL oxylipin composition that occur early in the development of cognitive decline, we identified non-MCI participants that exhibited reduced global cognitive function, categorized by an MoCA score <26 (68). We used mixed-model analysis of variance to assess grouped HDL oxylipin concentration differences based on participant cognitive status (non-MCI with normal MoCA, non-MCI with abnormal MoCA, or MCI with abnormal MoCA), gender, and race/ethnicity. Participant demographic information according to the three cognitive status groups can be found in Supplemental Table S1. We found significant interactions between cognitive status and gender (Fig. 6A, *P* = 0.0007) and between cognitive status and race/ethnicity (Fig. 6B, *P* = 0.001) on LOX-derived EPA C20:5ω3-derived HEPE concentrations. Non-MCI men with abnormal MoCA scores had higher EPA C20:5ω3-derived HEPE concentrations than MCI men with abnormal MoCA scores (Fig. 6A, *P* < 0.0001). Similarly, non-MCI Hispanic participants with abnormal MoCA scores had higher EPA C20:5ω3-derived HEPE concentrations than MCI Hispanic participants with abnormal MoCA scores (Fig. 6B, *P* < 0.0001).

### Correlations between participant HDL oxylipin concentrations and global cognitive function: participants with higher alcohol concentrations in HDL had higher cognitive function

Lastly, we used Spearman rank correlation to explore the relationship between grouped HDL oxylipin concentrations and continuous MoCA scores. We found that participant HDL alcohol concentrations were positively correlated with MoCA scores, particularly for non-Hispanic white women and ω3 oxylipins (Table 2). Concentrations of EPA C20:5ω3- and DHA C22:6ω3-derived alcohols made via the LOX enzymatic pathway of production in non-Hispanic white women were most strongly positively correlated with participant MoCA score (Table 2, *P* < 0.0001). Correlations between individual oxylipin concentrations and MoCA scores separated by gender and collapsed across race/ethnicity can be found in Supplemental Table S2.

**Table 2:** HDL alcohol concentrations positively correlated with MoCA scores

Gender	Race/ethnicity	Omega	PUFA	Chemistry	Pathway	Spearman ρ	*P*
W	Non-Hispanic White	3	EPA	Alcohol	LOX	0.326	<0.0001
	Non-Hispanic White	3	DHA	Alcohol	LOX	0.326	<0.0001
	Non-Hispanic White	3	DHA	Alcohol	CYP	0.356	0.0001
	Non-Hispanic White	6	LA	Alcohol	LOX	0.329	0.0004
	Non-Hispanic Black	3	EPA	Alcohol	LOX	0.145	0.004
	Non-Hispanic White	6	AA	Alcohol	LOX	0.181	0.006
M	Hispanic	3	DHA	Alcohol	LOX	0.436	0.0001
	Hispanic	6	LA	Alcohol	LOX	0.808	0.0002

The table lists significant Spearman rank correlations that passed false discovery rate correction at q = 0.1 between grouped HDL oxylipin concentrations and MoCA scores for the 222 participants included in this study. Participant MoCA scores ranged from 13 to 30.

AA, arachidonic acid; DHA, docosahexaenoic acid; EPA, eicosapentaenoic acid; LA, linoleic acid; M, men; W, women.

## Discussion

In this study, we found an association between MCI and lower concentrations of anti-inflammatory and vasodilatory ω3 oxylipins within HDL. We predicted that MCI would be associated with a shift toward more pro-inflammatory HDL oxylipin profiles, and the observed loss of anti-inflammatory oxylipins within HDL in MCI participants could contribute to an overall pro-inflammatory state within the body through impaired or reduced HDL anti-inflammatory effects. However, our results represent a snapshot in time of the highly dynamic process of HDL oxylipin metabolism, and more work is needed to elucidate the biological implications of our finding of an association between MCI and lower HDL concentrations of EPA C20:5ω3-derived HEPEs and DHA C22:6ω3-derived HDoHEs. It is possible that the observed loss of protective HDL biological effectors in MCI participants could exacerbate the known inverse relationship between lower plasma HDL-c concentrations and higher MCI risk (23, 92). Importantly, we found that HDL oxylipin profile differences between non-MCI and MCI participants were dependent on gender and race/ethnicity and were independent of plasma HDL-c concentrations. Whereas men and Hispanic individuals generally have lower plasma HDL concentrations than women and non-Hispanic white/Black individuals, respectively, plasma HDL-c concentrations in men and Hispanic individuals did not differ based on MCI status. In both men and Hispanic individuals with MCI, lower EPA C20:5ω3-derived HEPEs and DHA C22:6ω3-derived HDoHEs within HDL highlight an important gender- and race/ethnicity-specific dysregulated lipid pathway that may contribute to cognitive decline and may be a useful target for therapeutic development for these populations (39, 93). We further found in non-MCI participants with abnormal MoCA scores that higher concentrations of EPA C20:5ω3-derived HEPEs in HDL protected against the presence of MCI. In non-Hispanic white women, higher HDL oxylipin concentrations were associated with better cognitive function. Taken together, these findings indicate that there is a role for disrupted ω3 HDL oxylipin signaling during cognitive impairment in men and Hispanic individuals and identify a potential target for therapeutic intervention.

Our primary aim in this study was to determine how HDL oxylipin profiles differ by MCI status. We found that MCI status was associated with altered HDL oxylipin content and lower ω3 EPA C20:5ω3-derived HEPE and DHA C22:6ω3-derived HDoHE concentrations. These results corroborate other research findings showing lower concentrations of plasma EPA C20:5ω3- and DHA C22:6ω3-derived alcohols in individuals with AD compared to individuals without AD (57). Because HDL lipid composition affects HDL function and immunomodulatory properties, lower amounts of anti-inflammatory EPA C20:5ω3- and DHA C22:6ω3-derived oxylipins in HDL in participants with MCI may exacerbate neuroinflammation associated with MCI (94, 95). Previous research has found that *1*) higher PUFA intake reduces MCI and AD risk, *2*) EPA C20:5ω3 and DHA C22:6ω3 concentrations are lower in individuals with MCI and AD, *3*) EPA C20:5ω3 and DHA C22:6ω3 supplementation is associated with improved cognition in individuals with MCI, and *4*) brain EPA C20:5ω3 concentrations are positively correlated with cognitive function and negatively correlated with amyloid beta plaque and neurofibrillary tau tangle burdens in individuals with AD (30, 34, 96, 97, 98, 99, 100). The reduced concentrations of EPA C20:5ω3- and DHA C22:6ω3-derived oxylipins like 12-HEPE and 16-HDoHE, respectively, in the HDL of participants with MCI in our study may reflect lower availability of these precursor fatty acids, which maintain critical anti-inflammatory and vasodilatory functions in the brain (101). Future studies should assess how participant apoE genotype, medication history, and diet affect oxylipin profiles in individuals with or without MCI and whether individuals with MCI also have lower oxylipin concentrations in cerebrospinal fluid or postmortem brain tissue compared to non-MCI individuals. Overall, our finding of an association between a loss of anti-inflammatory ω3 HDL oxylipins and MCI provides a key insight into the molecular basis of how HDL and PUFA dysregulation contribute to cognitive impairment.

Oxylipin availability and biological activity are affected by precursor PUFA availability, the biosynthetic pathway of production, and functional group chemistry. Because of this, we explored how oxylipins that were grouped by these factors differed based on MCI status. We found that MCI was associated with lower concentrations of HDL alcohols synthesized by LOX, an emerging therapeutic target for AD (61). Ω3 LOX alcohols were significantly lower and ω6 LOX alcohols had a non-significant trend of being lower in the HDL of MCI participants compared to non-MCI participants. These results support a role for LOX enzymes in cognitive impairment and suggest that previously documented upregulation of LOX enzymes and LOX products in the brains of individuals with MCI and AD could be partially due to impaired removal of LOX products by HDL, which affects cellular inflammatory responses (41, 102). Further, these findings support the disruption of specific anti-inflammatory lipid signaling pathways in individuals with cognitive impairment. We also found a trend for higher concentrations of some CYP-derived oxylipins in the HDL of MCI participants, though more work is needed to understand how this pathway may be altered in MCI.

Given the known aforementioned gender and racial/ethnic disparities in the prevalence of MCI and dementia, we examined how HDL oxylipin profiles differed by gender and race/ethnicity in participants with or without MCI (11). Consistent with other studies that have shown lower concentrations of plasma HDL-c, EPA C20:5ω3, and DHA C22:6ω3 in men compared to women, we found that men with MCI had lower amounts of EPA C20:5ω3-derived HEPEs and DHA C22:6ω3-derived HDoHEs in HDL compared to men without MCI (93, 103). We further observed oxylipin-omic differences in MCI participants based on participant race/ethnicity. Participants with MCI who were Hispanic had lower concentrations of EPA C20:5ω3-derived HEPEs and DHA C22:6ω3-derived HDoHEs in HDL compared to Hispanic individuals without MCI; this is in line with Hispanic individuals having a higher risk for MCI and dementia, lower HDL concentrations, and lower plasma EPA C20:5ω3 and DHA C22:6ω3 concentrations compared to non-Hispanic white individuals (11, 36, 39). Because our study had a small sample size of Hispanic participants, future studies with a larger sample size of Hispanic participants are needed to explore differences in oxylipin profiles of Hispanic Black and Hispanic white individuals compared to non-Hispanic individuals. These gender- and race/ethnicity-specific findings have implications for precision treatment strategies in individuals with cognitive impairment and dementia.

We also evaluated the relationship between the severity of cognitive impairment and HDL oxylipin-omes. Although not all people with MCI will develop dementia, people with lower total MoCA scores are more likely to progress to AD than people with higher scores (104). In men and Hispanic participants with lower MoCA scores, higher concentrations of anti-inflammatory EPA C20:5ω3-derived HEPEs may protect against the presence of MCI. In non-Hispanic white women, higher concentrations of ω6 and ω3 alcohol oxylipins in HDL correlated positively with cognition. While we found that higher abundance of anti-inflammatory EPA C20:5ω3- and DHA C22:6ω3-derived alcohols (HEPEs and HDoHEs, respectively) within plasma HDL was associated with higher MoCA scores, we also observed positive correlations between the amount of pro-inflammatory LA C18:2ω6- and AA C20:4ω6-derived alcohols (hydroxyoctadecadienoic acids [HODEs] and HETEs, respectively) within HDL and higher MoCA scores. While this last finding may seem counterintuitive, similar positive associations between MoCA scores and blood-based inflammatory markers have been reported in individuals with AD (105). Taken together, a loss of HDL oxylipin content may contribute to cognitive impairment and MCI, and higher concentrations of certain oxylipins may be protective against cognitive impairment.

In summary, this research demonstrates that HDL oxylipin profiles are different in participants with or without MCI. This suggests that HDL oxylipins may have a role in the development of cognitive impairment. We identified anti-inflammatory and vasodilatory oxylipins in HDL that were lower in MCI participants compared to non-MCI participants. Gender- and race/ethnicity-specific HDL oxylipin differences identified men and Hispanic individuals as subgroups of the population who may experience the greatest cognitive benefit from interventions like ω3 fatty acid supplementation. Future studies are needed to assess *1*) oxylipin-ome changes over time during the initiation and progression of cognitive impairment, *2*) the impact of apoE genotype on HDL oxylipin differences between individuals with or without MCI, and *3*) whether targeting specific oxylipin signaling pathways is useful for cognitive impairment therapeutic development. Our findings suggest that immunomodulatory oxylipins are a potential mechanistic link between HDL, inflammation, MCI, and ultimately dementia risk.

## Data availability

Data are available through reasonable request from the EAS (einsteinagingstudy.com).

## Supplemental data

This article contains supplemental data.

## Conflict of interest

Dr Gregory Shearer is a consultant for Inipharm, Inc. All other authors declare that they have no conflicts of interest with the contents of this article.
