# Supplementary material for: Oxylipin composition of high-density lipoprotein is altered in men and Hispanic adults with mild cognitive impairment
Source: J Lipid Res. 2026 Mar 12;67(4):101021. doi: 10.1016/j.jlr.2026.101021 (PMC13092600; doi:10.1016/j.jlr.2026.101021)
Supplement: Supplementary Figures Tables [file mmc1.docx]

**Supplementary Figure 1: Participants with mild cognitive impairment (MCI) had lower oxylipin concentrations in high-density lipoprotein (HDL).** Graph indicates the nanomolar (nM) oxylipin concentrations with the 95% confidence interval (CI) of global total (non-esterified + esterified) oxylipins in HDL in individuals with or without MCI. *******P*<0.01.

**Supplementary Figure 2: Participants with mild cognitive impairment (MCI) had lower concentrations of anti-inflammatory eicosapentaenoic (EPA) C20:5**ω**3/lipoxygenase (LOX)-derived hydroxy-eicosapentaenoic acids (HEPEs) in high-density lipoprotein (HDL).** Graph indicates the nanomolar (nM) oxylipin concentrations with the 95% confidence interval (CI) of total (non-esterified + esterified) oxylipins in HDL in individuals with or without MCI. *******P*<0.01.

**Supplementary Figure 3: Men and Hispanic participants with mild cognitive impairment (MCI) had lower concentrations of anti-inflammatory omega (**ω**)3 oxylipins in high-density lipoprotein (HDL).** Graphs indicate the nanomolar (nM) oxylipin concentrations with the 95% confidence interval (CI) of total (non-esterified + esterified) eicosapentaenoic (EPA) C20:5ω3/lipoxygenase (LOX)-derived hydroxy-eicosapentaenoic acids (HEPEs) **(A)** and docosahexaenoic acid (DHA) C22:6ω3/LOX-derived hydroxy-docosahexaenoic acids (HDoHEs) in HDL in individuals with or without MCI. W: women. M: men.

| **Cognitive Status** | **Non-MCI, Normal MoCA: 73 (34%)** | **Non-MCI, Abnormal MoCA: 86 (40%)** | **MCI, Abnormal MoCA: 55 (26%)** |
| --- | --- | --- | --- |
| **Women** | 50 (68.5%) | 56 (65.1%) | 33 (60.0%) |
| **Non-Hispanic Black** | 23 (31.5%) | 36 (41.8%) | 28 (50.9%) |
| **Hispanic** | 3 (4.1%) | 14 (16.3%) | 12 (21.8%) |
| **Non-Hispanic White** | 47 (64.4%) | 36 (41.9%) | 15 (27.3%) |
| **Age (Years)** | 76.9 (75.8, 78.0) | 77.1 (76.1, 78.1) | 77.7 (76.5, 79.0) |
| **MoCA** | 27.3 (26.8, 27.8)*^A^* | 22.4 (21.9, 22.9)*^B^* | 20.3 (19.7, 20.9)*^C^* |
| **Education (Years)** | 16.8 (16.1, 17.6)*^A^* | 14.1 (13.4, 14.8)*^B^* | 14.0 (13.1, 14.9)*^B^* |
| **GDS** | 1.93 (1.47, 2.39) | 2.09 (1.67, 2.52) | 2.75 (2.22, 3.28) |

**Supplementary Table 1: Demographics of study participants grouped by mild cognitive impairment (MCI) status and Montreal Cognitive Assessment (MoCA) scores.** The number of participants for each cognitive status is listed in Supplementary Table 1, with the proportion of each group relative to the total study sample size shown in parentheses. The number of participants of each gender and ethnicity/race are similarly displayed, with the proportion of each group relative to their respective cognitive status group shown in parentheses. Participant mean age in years, Montreal Cognitive Assessment (MoCA) score, education in years, and Geriatric Depression Scale (GDS) score are listed with the 95% confidence interval shown in parentheses. A MoCA score of 26 or above was considered normal. Significant differences are indicated by differing superscript letters.

| **Gender** | **Ω** | **pFA** | **Chemistry** | **Pathway** | **Oxylipin** | **Spearman ρ** | ***P* value** |
| --- | --- | --- | --- | --- | --- | --- | --- |
| **W** | **3** | **DHA** | **Alcohol** | **CYP** | **22-HDoHE** | **0.263** | **0.0014** |
| W | 3 | EPA | Alcohol | LOX | 5-HEPE | 0.238 | 0.0039 |
| **W** | **6** | **AA** | **Diol** | **LOX** | **5,12-DiHETE** | **0.236** | **0.0045** |
| **W** | **3** | **EPA** | **Alcohol** | **LOX** | **12-HEPE** | **0.228** | **0.0058** |
| W | 3 | DHA | Alcohol | LOX | 13-HDoHE | 0.206 | 0.0127 |
| W | 6 | LA | Alcohol | LOX | 13-HODE | 0.201 | 0.0151 |
| W | 3 | DHA | Alcohol | CYP | 20-HDoHE | 0.189 | 0.0232 |
| W | 6 | AA | Alcohol | LOX | 9-HETE | 0.188 | 0.0233 |
| W | 3 | DHA | Alcohol | LOX | 8-HDoHE | 0.193 | 0.0237 |
| W | 3 | EPA | Alcohol | CYP | 18-HEPE | 0.187 | 0.0246 |
| W | 3 | DHA | Alcohol | LOX | 7-HDoHE | 0.186 | 0.0248 |
| W | 3 | DHA | Alcohol | LOX | 10-HDoHE | 0.188 | 0.0248 |
| W | 3 | DHA | Epoxide | CYP | 7(8)-EpDPE | 0.187 | 0.0263 |
| W | 3 | DHA | Alcohol | LOX | 16-HDoHE | 0.182 | 0.0283 |
| W | 3 | EPA | Alcohol | LOX | 8-HEPE | 0.191 | 0.0294 |
| W | 3 | EPA | Alcohol | LOX | 9-HEPE | 0.181 | 0.0296 |
| W | 6 | AA | Alcohol | LOX | 12-HETE | 0.177 | 0.033 |
| W | 3 | EPA | Diol | CYP | 17,18-DiHETE | 0.175 | 0.0355 |
| W | 3 | EPA | Alcohol | LOX | 11-HEPE | 0.179 | 0.0365 |
| W | 6 | LA | Alcohol | LOX | 9-HODE | 0.167 | 0.0451 |

**Supplementary Table 2: Individual high-density lipoprotein (HDL) oxylipin concentrations in women positively correlated with Montreal Cognitive Assessment (MoCA) scores.** Spearman rank correlation was used to assess potential linear relationships between participant HDL oxylipin concentrations and MoCA scores (N=222). Possible MoCA scores were between 0 and 30, with 30 representing the best possible score for cognitive function. Correlations between participant HDL oxylipin concentration and MoCA score where *P*<0.05 are listed in Supplementary Table 2. Correlations that passed false discovery rate (FDR) correction where q=0.2 are found in bold. W: women. pFA: parent fatty acid. LOX: lipoxygenase. CYP: cytochrome p450. LA: linoleic acid. AA: arachidonic acid. EPA: eicosapentaenoic acid. DHA: Docosahexaenoic acid. HDoHE: hydroxy-docosahexaenoic acid. HEPE: hydroxy-eicosapentaenoic acid. DiHETE: dihydroxy-eicosatetraenoic acid. HODE: hydroxy-octadecadienoic acid. HETE: hydroxy-eicosatetraenoic acid. EpDPE: epoxy-docosapentaenoic acid.
