# Supplementary material for: Oxylipin composition of high-density lipoprotein is altered in men and Hispanic adults with mild cognitive impairment
Source: J Lipid Res. 2026 Mar 12;67(4):101021. doi: 10.1016/j.jlr.2026.101021 (PMC13092600; doi:10.1016/j.jlr.2026.101021)
Supplement: Supplementary Materials [file mmc2.docx]

**SUPPLEMENTARY METHODS**

HDL isolation: The method of HDL-rich plasma isolation from whole plasma using FPLC has been previously published several times^39,57,58,59^. Briefly, the FPLC column was conditioned with a degassed 0.9% sodium chloride (pH 7.4) mobile phase containing 0.1 g/L EDTA and 0.1 g/L butylated hydroxytoluene (BHT) antioxidants at a flow rate of 0.5 mL per minute for at least one hour prior to sample injection. Participant plasma samples (100-200 𝜇L) were centrifuged through a 0.22 𝜇M pore cellulose acetate membrane filter tube and injected on the conditioned FPLC column. Plasma lipoproteins were separated by size at the same 0.5 mL/minute flow rate and with the same degassed, antioxidant-containing mobile phase used to condition the column. HDL fractions (250 𝜇L per fraction) of FPLC-separated plasma eluted 31-32.5 minutes after plasma sample injection on the FPLC, pooled together, and immediately frozen at -80℃ until further processing for oxylipin extractions. HDL fractions of FPLC-separated plasma were determined according to previously published papers from the laboratory and additional confirmation of enrichment of HDL apolipoproteins (e.g. apoA-I, apoA-II) in eluted HDL fractions as determined by SDS-PAGE^39,57,58,59^.
